# Synergistically Enhanced Ta_2_O_5_/AgNPs SERS Substrate Coupled with Deep Learning for Ultra-Sensitive Microplastic Detection

**DOI:** 10.3390/ma19010090

**Published:** 2025-12-25

**Authors:** Chenlong Zhao, Yaoyang Wang, Shuo Cheng, Yuhang You, Yi Li, Xianwu Xiu

**Affiliations:** School of Physics and Electronics, Shandong Normal University, Jinan 250014, China

**Keywords:** Ta_2_O_5_, SERS, hydrothermal synthesis, nanoplastic detection, morphology engineering, deep learning

## Abstract

**Highlights:**

**What are the main findings?**

- Fabricated spherical Ta_2_O_5_/AgNPs substrates with pseudo-Neuston networks.
- Achieved an ultra-low detection limit of 10^−13^ M for R6G via EM/CM contribution.
- Developed a CNN-Transformer model achieving 98.7% accuracy in high-noise spectra.

**What are the implications of the main findings?**

- Provides a scalable strategy for enhancing semiconductor SERS activity.
- Overcomes spectral interference in complex environmental microplastic detection.
- Demonstrates deep learning’s potential in robust automated spectral analysis.

**Abstract:**

Herein, a high-performance Ta_2_O_5_/AgNPs composite Surface-Enhanced Raman Scattering (SERS) substrate is engineered for highly sensitive detection of microplastics. Through morphology modulation and band-gap engineering, the semiconductor Ta_2_O_5_ is structured into spheres and composited with silver nanoparticles (AgNPs), facilitating efficient charge transfer and localized surface plasmon resonance (LSPR). This architecture integrates electromagnetic (EM) and chemical (CM) enhancement mechanisms, achieving an ultra-low detection limit of 10^−13^ M for rhodamine 6G (R6G) with excellent linearity. Furthermore, the three-dimensional “pseudo-Neuston” network structure exhibits superior capture capability for microplastics (PS, PET, PMMA). To address spectral interference in simulated complex environments, a multi-scale deep-learning model combining wavelet transform, Convolutional Neural Networks (CNN), and Transformers is proposed. This model achieves a classification accuracy of 98.7% under high-noise conditions, significantly outperforming traditional machine learning methods. This work presents a robust strategy for environmental monitoring, offering a novel solution for precise risk assessment of microplastic pollution.

## 1. Introduction

Raman spectroscopy is a powerful analytical technique based on inelastic scattering associated with the band structure of materials. Due to distinct molecular structures, substances exhibit unique “fingerprint” spectra, enabling precise compositional and microstructural identification [1]. However, the inherently low cross-sections of native Raman scattering necessitate Surface-Enhanced Raman Scattering (SERS) for signal amplification [2]. SERS enhances signals by inducing localized surface plasmon resonance (LSPR) on specific nanostructures, typically achieving enhancement factors exceeding 10^7^, which significantly lowers detection limits [3,4]. SERS mechanisms are primarily categorized into electromagnetic enhancement (EM) and chemical enhancement (CM) [5]. EM enhancement stems from strong localized electromagnetic fields generated by noble-metal nanostructures under laser illumination, whereas CM enhancement amplifies Raman intensity through charge transfer mechanisms between the target molecules and the substrate. The synergy of these mechanisms expands the applicability of SERS and provides a theoretical basis for ultra-sensitive detection.

Initially, noble-metal materials garnered extensive attention due to their exceptional SERS performance [6,7,8]. However, despite their high enhancement factors, noble metals often suffer from high costs and instability in complex environments [9], prompting the exploration of alternative materials. To address these limitations, recent advancements have expanded towards label-free and metal-free SERS approaches that emphasize minimally invasive designs and multifunctionality. For instance, Keshavarz et al. [10] developed self-assembled nitrogen-doped carbon nanostructures as a metal-free SERS platform, successfully demonstrating both intracellular biomolecular monitoring and selective therapeutic functionality. Driven by these evolving requirements for stability and biocompatibility, semiconductor materials have emerged as promising candidates for next-generation SERS substrates, offering advantages such as controllable morphology, high thermal stability, and biocompatibility [11,12,13,14]. Among these, Tantalum pentoxide (Ta_2_O_5_) is widely utilized in optical and catalytic fields due to its superior stability against corrosion and extreme pH/temperature conditions [15]. Recent studies suggest that defect engineering, heterojunction construction, or element doping can create efficient charge transfer pathways on Ta_2_O_5_ surfaces, thereby boosting SERS activity [16,17,18,19].

Motivated by these properties, this study designed a spherical Ta_2_O_5_ and silver nanoparticle (AgNPs) composite substrate via morphological modulation and band-gap engineering, achieving the organic integration of EM and CM mechanisms. Unlike traditional single-component substrates, this composite leverages oxygen vacancies and Ta^4+^ defects on the Ta_2_O_5_ surface to improve interfacial charge transfer efficiency, significantly enhancing molecular adsorption and spectral responsiveness. Additionally, the hydrothermally self-assembled spherical Ta_2_O_5_ structures provide abundant “hotspots” for the directional deposition of AgNPs, forming high-density electromagnetic coupling regions. Experimental results demonstrate that the Ta_2_O_5_/Ag composite achieves a detection limit of 10^−13^ M for rhodamine 6G (R6G), exhibiting outstanding sensitivity and uniformity.

Notably, the fabricated architecture possesses a nanostructure resembling “pseudo-Neuston” nets, a morphology confirmed to enhance microplastic capture and SERS effects [20,21]. This three-dimensional structure, combining macropores and mesopores, amplifies the local electromagnetic field intensity and effectively adsorbs microplastics through capillary action and hydrophobic interactions, demonstrating substantial potential for environmental detection.

In practical microplastic detection, however, factors such as water impurities, thermal noise, and fluorescence often introduce interference signals like Gaussian white noise and baseline drift, causing spectral distortion [22]. To improve interpretation accuracy, machine learning methods (e.g., SVM, RF, LDA) have shown advantages over manual analysis [23]. While effective in low-noise scenarios, their performance degrades sharply under high-noise conditions. Consequently, deep learning approaches have gained traction [24,25,26]. Convolutional Neural Networks (CNNs) excel at extracting multi-channel spectral information even from small datasets [4,21,27,28]. However, the fixed receptive field of CNNs limits global feature extraction, and stacking layers increases computational complexity [29]. Conversely, Artificial Neural Networks (ANNs) model global features but struggle to recognize coupling effects among adjacent wavenumbers [30].

Recently, Transformer models based on self-attention mechanisms have demonstrated superior performance in handling long-range dependencies [31,32]. Increasing research employs Transformers for spectral identification due to their powerful global information processing capabilities. Integrating Transformers with CNNs has emerged as a robust strategy for analyzing complex spectral data [33].

Herein, we present a dual-optimization strategy involving material fabrication and model design. First, a low-cost SERS substrate combining spherical Ta_2_O_5_ and AgNPs was fabricated, achieving a detection limit of 10^−13^ M for R6G. Second, a fusion deep-learning model combining wavelet transform, CNN, and Transformer was proposed to precisely identify microplastics in high-noise scenarios. Comparative results indicate that this model maintains a classification accuracy above 98.7% under extreme noise conditions, validating the significant potential of the proposed system for environmental monitoring and risk assessment.

## 2. Materials and Methods

### 2.1. Materials

Tantalum pentoxide (Ta_2_O_5_, 99.99%), hydrofluoric acid (HF, 40%), hydrogen peroxide solution (H_2_O_2_, 30%), silver nitrate (AgNO_3_), and polyvinylpyrrolidone (PVP) were purchased from Aladdin Reagent Co., Ltd., (Shanghai, China). Acetone, ethylene glycol, ammonia solution, and absolute ethanol were supplied by Sinopharm Chemical Reagent Co., Ltd., (Shanghai, China). Polystyrene (PS), polymethyl methacrylate (PMMA), and polyethylene terephthalate (PET) were obtained from Hai’an Zhichuan Battery Materials Technology Co., Ltd., (Nantong, China). Deionized water used in all experiments was freshly prepared in-house.

### 2.2. Preparation of Silver

Nanoparticles Silver nanoparticles (AgNPs) were synthesized via a chemical reduction method [17,34,35]. Briefly, ethylene glycol was added to a round-bottom flask and magnetically stirred. PVP was added until the solution became transparent. Simultaneously, deionized water and AgNO_3_ were mixed in a test tube until completely dissolved. The round-bottom flask was rapidly heated in an oil bath to 120 °C, after which the prepared AgNO_3_ solution was added and stirred thoroughly. The mixture was maintained at this temperature for 40 min until the solution turned a characteristic yellow-brown color. After cooling, the product was washed with acetone and centrifuged. This washing cycle was repeated several times to obtain the final AgNPs solution.

### 2.3. Preparation of Ta_2_O_5_

Nanostructures Ta_2_O_5_ nanoparticles were synthesized via a two-step hydrothermal method. First, Ta_2_O_5_ powder was dissolved in HF and transferred into a PTFE-lined autoclave, reacting at 110 °C for 12 h. After cooling to room temperature, ammonia solution was added to adjust the pH to approximately 9, yielding a white precipitate. The precipitate was collected, washed, and dried to obtain the Ta_2_O_5_ precursor. Subsequently, the precursor was dissolved in a mixed solution of H_2_O_2_ and ammonia under stirring. HF was slowly added, and the mixture underwent hydrothermal treatment at 240 °C for 24 h. Finally, a white suspension containing Ta_2_O_5_ nanoparticles was obtained after thorough washing.

### 2.4. Preparation of the Composite Substrate

**Figure 1:**
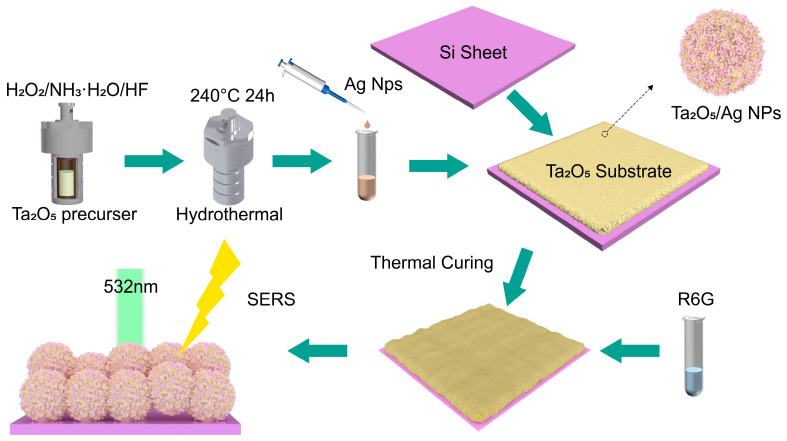
Schematic illustration of the preparation process for the Ta_2_O_5_/AgNPs composite SERS substrate.

The preparation process is illustrated in Figure 1. First, both the AgNPs and Ta_2_O_5_ nanoparticle solutions were ultrasonicated for 30 min to ensure homogeneous dispersion. The Ta_2_O_5_ and AgNP solutions were then mixed thoroughly at a volume ratio of 2:1. A distinct volume of the mixture was drop-cast onto a clean silicon wafer. The substrate was heated to facilitate solidification, yielding the Ta_2_O_5_/AgNPs composite SERS substrate.

### 2.5. Data Acquisition Method

Rhodamine 6G (R6G) was selected as the probe molecule to quantitatively evaluate SERS performance [36]. R6G solutions were prepared with concentrations ranging from 10^−7^ M to 10^−13^ M. Specifically, 10 μL of each analyte solution was drop-cast onto the substrate and dried on a heating stage at 50 °C. For microplastic detection, three types of microplastics were diluted and applied following the same procedure. Raman spectra were acquired using a 532 nm excitation laser with a power of 0.48 mW and an integration time of 4 s.

### 2.6. Characterization and Instruments

Morphologies were characterized using a scanning electron microscope (SEM, Gemini Sigma 500, ZEISS, Oberkochen, Germany). Elemental composition was analyzed via energy-dispersive X-ray spectroscopy (EDS, QUANTAX, Bruker, Billerica, MA, USA). Optical absorption spectra and bandgaps were determined using a UV–visible spectrophotometer (Alpha-1500, Shanghai Mapada, Shanghai, China). Surface chemical states were investigated using X-ray photoelectron spectroscopy (XPS, Escalab 250Xi, Thermo Scientific, Waltham, MA, USA). Raman measurements were performed using a Raman spectrometer (HR Evolution 800, Horiba, Kyoto, Japan). Spectra were collected from 10 randomly selected points to calculate the average signal.

## 3. Results and Discussion

### 3.1. Characterization of Ta_2_O_5_ Nanostructures and Composite Substrates

**Figure 2:**
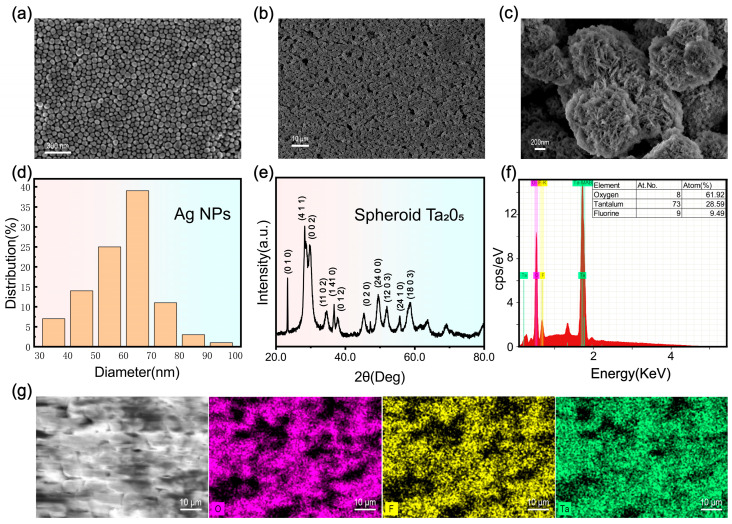
(**a**–**c**) SEM images of silver nanoparticles and spherical Ta_2_O_5_; (**d**) size distribution of silver nanoparticles; (**e**) XRD pattern of spherical Ta_2_O_5_; (**f**,**g**) element mapping (O, F, Ta) of spherical Ta_2_O_5_.

SEM images reveal that the synthesized AgNPs exhibit a uniform spherical morphology with a narrow size distribution, predominantly ranging from 50 to 70 nm (Figure 2a,d). This size range (60–70 nm) is reported to be optimal for maximizing the localized surface plasmon resonance (LSPR) effect in SERS applications [37].

The hydrothermally synthesized F-Ta_2_O_5_ nanostructures manifest as uniform microspheres with an average diameter of approximately 1 μm (Figure 2b,c). High-magnification observations indicate that these spheres are hierarchical structures composed of aggregated fine nanorods, consistent with the typical growth behavior of Ta_2_O_5_ [38]. Typically, Ta_2_O_5_ tends to grow anisotropically along the (010) crystal plane [38,39,40]. However, in this study, the introduction of fluoride ions (F^−^) played a critical regulating role. F^−^ ions selectively adsorb onto high-energy facets (e.g., (010)), effectively inhibiting longitudinal elongation along this axis and promoting multi-directional nucleation. Consequently, the nanorods self-assemble into spherical aggregates rather than forming long wires.

Crucially, compared to commercial Ta_2_O_5_, these synthesized hierarchical spheres possess significantly higher surface roughness at both the micro- and nanoscale. This distinctive topography creates abundant interstitial voids, allowing AgNPs to spontaneously embed and form a tightly coupled Ta_2_O_5_/AgNPs hybrid interface. Such a structure is pivotal for creating high-density electromagnetic “hotspots” and enhancing molecular capture efficiency.

To further support this morphology-driven hotspot interpretation, we performed finite-element-method (FEM) simulations at 532 nm using representative geometries simplified from the SEM observations. In the model, the Ta_2_O_5_ surface texture was approximated by nanorod-like features (120 nm × 10 nm) forming concave interstitial voids, and Ag nanoparticles were set to 60 nm in diameter. The simulated near-field maps (Figure S8) show that the local field enhancement $\frac{\left|E\right|}{\left|E_{0}\right|}$ is strongly localized at Ag–Ag nanogaps and Ag–Ta_2_O_5_ junction regions, and the composite configuration exhibits a higher peak value and broader hotspot distribution $\frac{\left|E\right|}{\left|E_{0}\right|}$ (≈29.2) than the AgNP-only configuration (≈13.8), supporting that the Ta_2_O_5_-assisted geometry promotes stronger field confinement and a higher density of effective hotspots.

X-ray diffraction (XRD) analysis confirms the high crystallinity of the hydrothermally synthesized Ta_2_O_5_, indexing well to the orthorhombic β-phase (JCPDS No. 79-1375) [41] (Figure 2e). Dominant peaks at 22.9°, 28.3°, and 29.5° are assigned to the (010), (410), and (002) planes, respectively, indicating preferential growth orientation. Additional reflections (e.g., at 34.1° and 36.3°) further attest to the structural integrity of the sample.

Energy-dispersive X-ray spectroscopy (EDS) mapping demonstrates a uniform elemental distribution across the porous architecture (Figure 2g). Quantitatively, the atomic ratio of O:Ta is calculated to be approximately 2:1 (Figure 2f), which is notably lower than the stoichiometric 2.5:1 ratio of standard Ta_2_O_5_. This oxygen deficiency is attributed to the generation of abundant oxygen vacancies and Ta^4+^ species during the hydrothermal reduction process, a defect structure critical for enhancing charge transfer as verified by subsequent XPS analysis.

**Figure 3:**
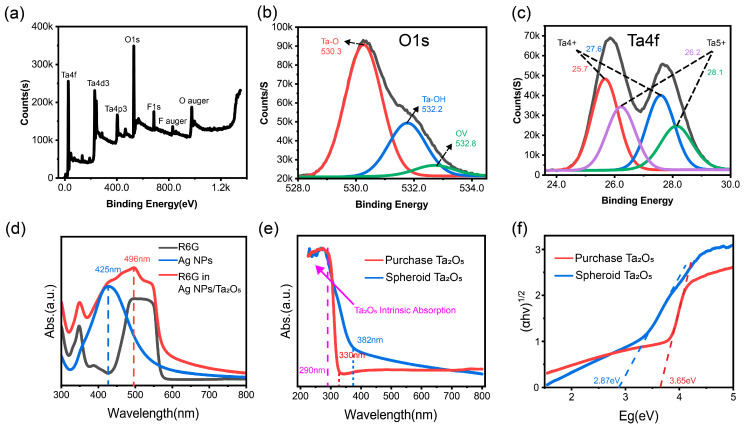
(**a**–**c**) XPS survey, O 1s, and Ta 4f spectra; (**d**) UV-Vis spectra of various substrates; (**e**,**f**) Absorbance spectra and Tauc plots comparing synthesized and commercial Ta_2_O_5_. The gray lines in (**b**,**c**) represent the experimental data (raw spectra).

To further investigate the chemical composition and electronic structure, XPS measurements were conducted on the spherical Ta_2_O_5_ (Figure 3a). No detectable impurity peaks were observed, indicating high sample purity. The Ta 4f spectrum (Figure 3c) was deconvoluted into four peaks: the doublets at 26.2 eV and 28.1 eV correspond to Ta^5+^ 4f_5_/_2_ and 4f_7_/_2_, while the peaks at 25.7 eV and 27.6 eV correspond to Ta^4+^ 4f_5_/_2_ and 4f_7_/_2_, respectively. The coexistence of Ta^5+^ and Ta^4+^ components is widely considered indicative of lattice oxygen deficiency (oxygen-vacancy-related defects) in Ta_2_O_5_.

The O 1s spectrum (Figure 3b) exhibited a main peak at 530.3 eV, corresponding to Ta–O bonds in Ta_2_O_5_, indicating that the material is primarily composed of tantalum oxide. A secondary peak at 531.7 eV was attributed to surface Ta–OH groups, likely arising from adsorbed water or surface hydroxylation. An additional peak at 532.8 eV was also detected and is defect-related oxygen in an oxygen-deficient environment (VO-associated species), based on literature comparisons and the presence of Ta^4+^ signals in the Ta 4f spectrum. The introduction of oxygen vacancies alters the local chemical environment, redistributing electron density and resulting in an upward shift in binding energy, supported by the Ta^4+^ signature in Ta 4f. Because XPS is surface sensitive, a minor contribution from adsorbed oxygen-containing species cannot be fully excluded. Notably, no Si 2p/2s signal is observed in the survey spectrum (Figure 3a), making O–Si-related contributions unlikely.

As shown in Figure 3d, UV–Vis absorption spectra were collected to support the excitation-wavelength selection. Bare AgNPs exhibit a localized surface plasmon resonance (LSPR) peak centered at ~425 nm. To probe the practical optical response of the analyte–substrate interaction system, the spectrum was recorded using the Ta_2_O_5_/AgNPs composite powder dispersed in an R6G solution with a concentration of 10^−3^ M. Compared with bare AgNPs, the R6G–Ta_2_O_5_/AgNPs mixture displays a broadened absorption profile with a pronounced feature around ~496 nm and an absorption shoulder extending toward the 532 nm region. Although high loading and possible dye aggregation at this concentration can produce a plateau-like band shape, the appearance of the ~496 nm feature together with the shoulder near 532 nm indicates that the optical response of R6G is modified in the presence of the composite interface. Therefore, a 532 nm laser was selected for subsequent SERS measurements to operate under resonance/pre-resonance conditions of the dye in the composite environment and to achieve strong and reproducible SERS signals.

Figure 3e shows that, compared to the commercial Ta_2_O_5_ (with an absorption edge at approximately 330 nm), the synthesized Ta_2_O_5_ substrate exhibits enhanced absorption beyond 382 nm in the ultraviolet region, with a significantly broadened absorption band. This shift indicates a narrower bandgap in the synthesized Ta_2_O_5_. Based on Tauc plot analysis derived from the absorption spectrum (Figure 3f), the bandgap of the synthesized Ta_2_O_5_ was calculated to be approximately 2.87 eV, whereas that of the commercial sample was wider, at around 3.65 eV. The reduction in bandgap is primarily attributed to the formation of a high concentration of Ta^4+^ species and oxygen vacancies during the synthesis process, which is consistent with previous reports on hydrothermally synthesized Ta_2_O_5_ nanostructures [41].

The incorporation of oxygen vacancies introduces defect states into the band structure, modifying the electronic configuration of the material and resulting in a substantial narrowing of the bandgap. This structural feature not only enhances the substrate’s light-harvesting capabilities but also opens new possibilities for its application in optical and electronic devices.

### 3.2. Raman Results

**Figure 4:**
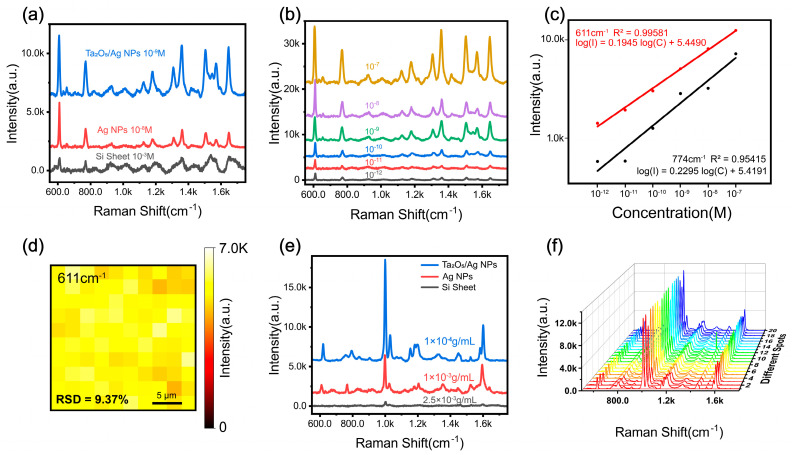
(**a**) Raman spectra of R6G on the Ta_2_O_5_/AgNPs composite, pure AgNPs, and bare silicon substrates; (**b**) SERS spectra of R6G on the composite substrate at different concentrations (10^−7^–10^−12^ M); (**c**) Linear fitting of the Raman intensity versus the logarithm of R6G concentration at 611 cm^−1^ and 774 cm^−1^; (**d**) SERS mapping image of R6G on the composite substrate; (**e**) Raman spectra of PS on the composite substrate, AgNPs, and bare silicon; (**f**) SERS mapping of PS intensity distribution at different sampling points.

To comprehensively evaluate the SERS activity, the Raman response of R6G on AgNPs, the composite substrate (Ta_2_O_5_/AgNPs), commercial Ta_2_O_5_, and bare silicon wafers was compared (Figure 4a). Results demonstrate that the composite substrate exhibits the most pronounced signal enhancement, with peak intensities significantly surpassing those of pure AgNPs. The limit of detection (LOD) for R6G on the composite substrate reaches as low as 10^−13^ M (Figure S1). This high sensitivity can be rationalized by the combined contributions of EM enhancement from AgNPs and a defect-associated CM contribution from the Ta_2_O_5_ support. Specifically, the high density of oxygen vacancies on the Ta_2_O_5_ surface, as revealed by XPS, creates additional active sites and modifies the electronic band structure, thereby facilitating efficient charge transfer between the substrate and analyte molecules.

Furthermore, the quantitative detection capability was assessed by analyzing the correlation between Raman intensity and R6G concentration (Figure 4b). The characteristic peaks at 611 cm^−1^ and 774 cm^−1^ exhibit strong linear relationships with the logarithm of the R6G concentration (Figure 4c), yielding correlation coefficients $R^{2}$ of 0.996 and 0.954, respectively. Statistical analysis yielded *p*-values < 0.001 for both peaks, confirming the significance of the linear correlation. These high linearity values provide a reliable basis for quantitative analysis. Raman mapping of the 611 cm^−1^ peak further confirmed the spatial uniformity of the substrate across the tested area (Figure 4d), which is attributed to the homogeneous dispersion of Ta_2_O_5_ and AgNPs achieved during ultrasonication. This excellent spatial uniformity ensures high reproducibility and reliability in practical SERS measurements.

The uniformity and reproducibility of the substrate were evaluated by calculating the relative standard deviation (RSD) of the SERS signal. Six batches of Ta_2_O_5_/AgNPs composite substrates were prepared using R6G (10^−10^ M) as the probe molecule. For each substrate, five measurement points were randomly selected, yielding a total of 30 spectral measurements. The RSD of the characteristic peak at 611 cm^−1^ was calculated using Equation (1):

(1) $$RSD=\frac{S}{\bar{I}}\times 100\%=\frac{\sqrt{\frac{1}{n-1}\sum_{i=1}^{n}{\left(I_{i}-\bar{I}\right)}^{2}}}{\bar{I}}$$

where $I_{i}$ is the SERS signal intensity at 611 cm^−1^ for each point, and $\bar{I}$ is the average intensity. The calculated RSD value is approximately 9.37%, demonstrating the excellent uniformity and batch-to-batch reproducibility of the Ta_2_O_5_/AgNPs substrate. To quantitatively assess the sensitivity, the analytical enhancement factor (AEF) for R6G was calculated using Equation (2):

(2) $$AEF=\frac{I_{SERS}}{I_{{SiO}_{2}}}\times \frac{C_{{SiO}_{2}}}{C_{SERS}}$$

In Equation (2), $I_{SERS}$ and $I_{{SiO}_{2}}$ are the intensities at 611 cm^−1^ measured on the Ta_2_O_5_/AgNPs substrate and on the bare SiO_2_ substrate, respectively, acquired under identical optical settings (objective, laser power, integration time, and an estimated spot size). $C_{SERS}$ and $C_{{SiO}_{2}}$ are the corresponding R6G concentrations. Because the exact number of molecules within the effective sampling volume after drop-casting/drying cannot be rigorously determined (possible drying-induced inhomogeneity), the value reported here should be regarded as an approximate AEF rather than an absolute single-molecule EF, the peak intensity reached 1403. Based on this, the AEF of the Ta_2_O_5_/AgNPs composite substrate was calculated to be approximately 2.33 × 10^9^.

**Figure 5:**
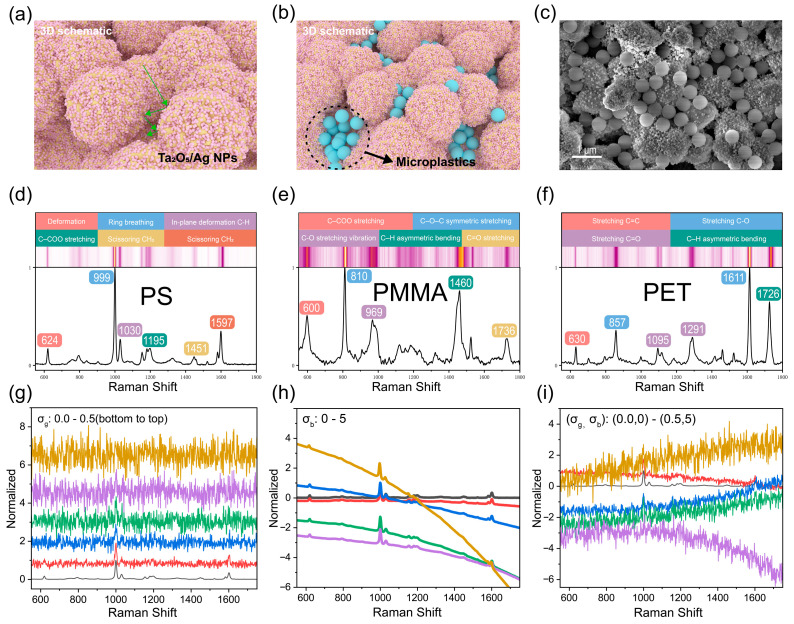
(**a**) 3D schematic illustration illustrating the proposed multiple/random light-scattering effect of the hierarchical spherical Ta_2_O_5_ architecture, (**b**) 3D schematic illustration showing the proposed microplastic trapping/encapsulation within the porous Ta_2_O_5_/AgNPs (“pseudo-Neuston”) architecture and the formation of volumetric hotspot regions around trapped particles. Panels (**a**,**b**) are conceptual renderings for visualization only and are not experimental micrographs. (**c**) SEM image of Ag–spherical Ta_2_O_5_–microplastic; (**d**–**f**) spectral profiles and characteristic Raman peaks of PS, PMMA, PET; (**g**–**i**) spectra with added Gaussian noise, baseline noise, and mixed noise. The green arrows in (**a**) illustrate the multiple light scattering paths. The numbers in (**d**–**f**) indicate the characteristic Raman peak positions (cm^−1^). For (**g**–**i**), the curves correspond to the parameter values listed in the legends, arranged from bottom to top.

Subsequently, the SERS capability for microplastic detection was evaluated (Figure 4e). Results indicate that the Ta_2_O_5_/AgNPs substrate produces significantly stronger Raman signals compared to conventional noble metal-based substrates. Mechanistically, this hierarchical porosity overcomes the limitations of traditional planar substrates. As shown in Figure S2, pure AgNPs exhibit poor affinity towards microplastics due to electroneutrality, often requiring external aggregating agents (e.g., NaCl, NaI) to force contact [42,43]. In contrast, our ‘pseudo-Neuston’ architecture employs a physical trapping strategy. During solvent evaporation, the mesopores generate strong capillary forces that actively drag the suspended microplastics into the interstitial voids [20]. Furthermore, the 3D encapsulation creates volumetric hotspots around the trapped particles [21], ensuring high signal reproducibility without chemical additives. This trapping/encapsulation process and the formation of volumetric hotspot regions are schematically illustrated in Figure 5b and are experimentally supported by SEM observations showing particles located within the porous pockets (Figure 5c).

Notably, we did not directly measure BET surface area or quantitative wetting/wicking kinetics (e.g., contact angle); therefore, the discussion of capillary-assisted trapping is presented as a mechanistic rationale consistent with the observed porous morphology and SEM evidence, rather than a quantitative comparison based on capillary metrics.

To define the detection limit for microplastics, we adopted a mass-concentration-based criterion due to the relatively large particle size and the stochastic particle occupancy within the laser spot. Here, the detection limit is defined as the lowest concentration at which characteristic Raman peaks of the target polymer can be consistently identified across multiple randomly selected points. Under our current sample-preparation and deposition protocol, the practical detection limit on the Ta_2_O_5_/AgNPs substrate is 1 × 10^−4^ g mL^−1^ (Figure 4e). At lower concentrations, the spectra become non-reproducible, showing pronounced point-to-point fluctuations and occasional spurious peaks, which we attribute to sparse particle distribution/settling and insufficient particle–hotspot contact within the probed volume. Therefore, 1 × 10^−4^ g mL^−1^ is reported as the operational LOD for reliable identification in this study.

To quantitatively characterize the ‘pseudo-Neuston’ network, the pore size distribution was analyzed based on SEM images using ImageJ software (v1.53k). As shown in Figure S3, the substrate exhibits a hierarchical pore distribution ranging primarily from 500 nm to 2000 nm, with an average pore size of approximately 1.15 ± 0.52 μm. These voids create dense SERS-active regions at the Ta_2_O_5_/AgNPs interfaces, enabling direct contact with analyte particles and significantly enhancing signal collection efficiency via LSPR effects. SEM imaging (Figure 5c) confirms that microplastic particles successfully fill these voids, a critical factor for the observed enhancement.

Additionally, the hierarchical surface roughness and interconnected void structure are expected to promote multiple/random scattering of the incident laser, thereby increasing the optical path length and the photon–analyte interaction probability within the 3D architecture. This scattering-assisted mechanism is schematically illustrated in Figure 5a (3D rendering) and is experimentally supported by the measured scattering intensity shown in Figure S4, which confirms the enhanced scattering capability of the Ta_2_O_5_-based substrate. Such 3D enhancement architectures have proven highly effective for microplastic detection [20,44].

Substrate uniformity was further corroborated by spectral measurements across different regions (Figure 4f). The consistent signal enhancement confirms the high spatial uniformity and reliability of the composite.

In summary, comparative analysis concludes that the Ta_2_O_5_/AgNPs composite exhibits the highest SERS intensity and lowest detection limit among the tested materials, consistent with the combined effects of dense plasmonic hotspots, hierarchical roughness/porosity, and defect-associated interfacial charge-transfer pathways.

### 3.3. Data Acquisition and Processing

To simulate realistic aquatic environments, three representative microplastics—polystyrene (PS), polymethyl methacrylate (PMMA), and polyethylene terephthalate (PET)—were selected as target analytes. Their characteristic Raman profiles are presented in Figure 5d–f, with specific peak assignments referenced from the literature [45]. To mitigate noise and reveal intrinsic spectral features, spectral smoothing was applied using a Savitzky–Golay (SG) filter with a window size of 11. Comparative heatmaps of unsmoothed versus smoothed spectra visually demonstrate the denoising efficacy. Notably, the Raman peaks of PS appear more prominent and less noisy compared to those of PET and PMMA, indicating that the Ta_2_O_5_/AgNPs composite substrate provides superior SERS enhancement specifically for PS.

To simulate the complex noise conditions encountered in real-world experimental environments, Gaussian White Noise (AWGN) (Figure 5g) and Baseline Drift Noise (BBN) (Figure 5h) were superimposed onto the normalized SERS spectra according to Equation (3). This process generated spectral datasets containing AWGN, BBN, and a combination of both, designated as Mixed Noise (MN) (Figure 5i).

(3) $$\tilde{S}\left(\lambda \right)=\frac{S\left(\lambda \right)}{\max S\left(\lambda \right)}+\sigma_{g}\cdot N\left(0,1\right)+\sigma_{b}\cdot B\left(\lambda \right)$$

Specifically, AWGN was generated by sampling from a standard Gaussian distribution, with its intensity modulated by a percentage factor ($\sigma_{g}$) to precisely simulate random spectral fluctuations. Baseline drift (BBN) was modeled using random polynomial functions ($\sigma_{b}\cdot B\left(\lambda \right)$) to mimic background variations typical of instrumental or environmental instability. To construct the final training dataset, maximum intensities of both AWGN and BBN were applied, as illustrated in the preceding figures. This configuration maximizes classification difficulty, thereby facilitating a rigorous evaluation of the model’s robustness and accuracy under extreme noise conditions.

### 3.4. Model Architecture

**Figure 6:**
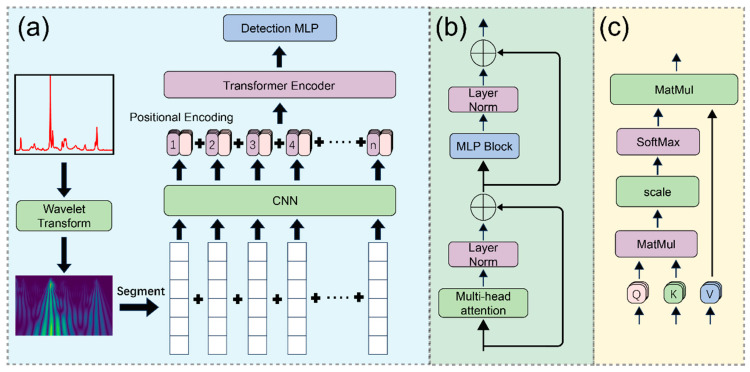
(**a**) Overall architecture of the data preprocessing and deep learning model. (**b**) Structure of the Transformer encoder. (**c**) Structure of the multi-head attention mechanism.

Figure 6 illustrates the proposed hybrid neural network architecture, which synergistically integrates the denoising capabilities of the Wavelet Transform, the local feature extraction strength of Convolutional Neural Networks (CNNs), and the global dependency modeling of Transformers (Figure 6a). Initially, noisy spectral data are decomposed into multi-channel representations via the wavelet transform. These multi-channel spectra are subsequently processed by a CNN to extract high-level spectral features. Positional encodings are then injected into the feature maps to retain sequence order information before feeding them into the Transformer encoder. Finally, the classification output is generated through a Multi-Layer Perceptron (MLP) head.

The encoder architecture, detailed in Figure 6b, comprises stacked attention blocks and MLP blocks connected via residual connections. The core attention mechanism, depicted in Figure 6c and mathematically defined by Equation (4), enables the model to dynamically weigh and focus on informative spectral regions across the entire sequence.

(4) $$Attention\left(Q,K,V\right)=softmax\left(\frac{QK^{T}}{\sqrt{d_{k}}}\right)V$$

Specifically, the dot product is computed between the query (Q) and key (K) matrices, scaled by the dimension factor ($d_{k}$), and normalized via a softmax function to obtain the attention weights. These weights are then applied to the value (V) matrix to synthesize the context-aware output. Detailed hyperparameters of the model architecture are provided in Table S1.

### 3.5. Model Evaluation

For each microplastic spectral class, 100 spectra were initially collected. To enhance model generalization, data augmentation was performed by superimposing varying levels of Gaussian and baseline noise, expanding the dataset tenfold. The augmented dataset was partitioned into training, validation, and testing sets with a ratio of 7:2:1. The training set was utilized for model fitting, the validation set for hyperparameter tuning and preliminary assessment, and the test set for the final performance evaluation.

Performance was quantified using confusion matrices, classification accuracy, and F1-scores. The deep learning model was implemented using Python v3.9.20 and the PyTorch v2.5.1+cu124 framework. Traditional machine learning baselines, including Random Forest (RF), K-Nearest Neighbors (KNN), Linear Discriminant Analysis (LDA), and Support Vector Machine (SVM), were implemented using Scikit-learn.

### 3.6. Identification of Noisy Spectra

**Figure 7:**
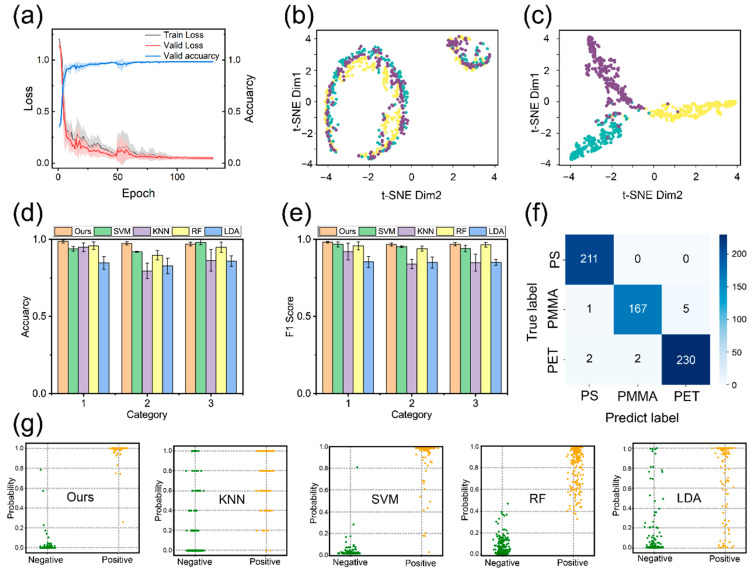
(**a**) Model training loss and accuracy curves; (**b**,**c**) t-SNE diagrams of original spectra vs. model output features; (**d**,**e**) accuracy and F1-score comparisons among different models; (**f**) confusion matrix for the proposed model; (**g**) probability distributions from model outputs.

The proposed model was trained on the noise-augmented dataset. Figure 7a illustrates the training dynamics, where solid lines and shaded areas represent the mean values and standard deviations over ten independent runs, respectively. In the initial 100 epochs, the model exhibited considerable fluctuations, attributed to the stochastic nature of weight initialization and the high noise levels. However, as training progressed, the loss values stabilized around epoch 130, facilitated by the introduction of learning rate decay. Beyond this point, both accuracy and loss reached a steady state.

The complete training dynamics are detailed in Figure S5. A close comparison of the training and validation loss curves reveals that they track each other closely, indicating that the model effectively avoids overfitting. Ultimately, the validation accuracy stabilized within the range of 97.8% to 99.1%, demonstrating the model’s robustness and superior performance in handling noisy spectral data.

To visualize the feature extraction capability, t-Distributed Stochastic Neighbor Embedding (t-SNE) [46] was applied (Figure 7b,c). In the raw spectral space, data points from different classes are heavily entangled due to noise interference, making classification challenging. In contrast, features extracted by the proposed model form distinct, compact clusters with minimal overlap. This separation demonstrates the model’s ability to effectively extract discriminative class-specific representations.

Comparative analysis (Figure 7d,e) highlights the superiority of the proposed deep learning architecture over traditional ML methods. While SVM achieved a respectable accuracy of 95.4% on denoised data, LDA performed poorly (84.6%) due to its sensitivity to boundary drift. Crucially, in the absence of denoising preprocessing, the performance of traditional models collapsed (Figure S6a,b). SVM accuracy plummeted to 39%, as its reliance on clear margins makes it vulnerable to raw spectral noise. In sharp contrast, our model, leveraging the multi-resolution features from Wavelet Transform and global context from Transformers, maintained an impressive accuracy of 98.7% directly on raw noisy spectra. Detailed class-wise performance metrics, including Precision and Recall, are further provided in Table S2.

The confusion matrix (Figure 7f) reveals minimal misclassification, aligning with the t-SNE results. Furthermore, to assess prediction confidence, the output layer was analyzed using probability distributions (Figure 7g). While SVM shows reasonably tight clustering, other traditional models fail to effectively distinguish between positive and negative samples without denoising (Figure S6c). Our model, however, produces sharply concentrated probability distributions, indicating high-confidence predictions. This robustness validates the model’s potential for deployment in complex, large-scale environmental monitoring tasks.

In summary, the comparative analysis confirms that the proposed model exhibits decisive advantages in noise immunity and feature discrimination, attributed to its adaptability to complex spectral data and capacity for deep feature integration.

## 4. Conclusions

In summary, this work presents a dual-optimization strategy integrating material engineering and deep learning for ultra-sensitive microplastic detection. A spherical Ta_2_O_5_/AgNPs composite substrate was successfully fabricated via hydrothermal self-assembly. By leveraging morphology modulation and band-gap engineering, the substrate benefits from combined electromagnetic (EM) and defect-associated chemical enhancement (CM) contributions, achieving an ultra-low detection limit of 10^−13^ M for rhodamine 6G (R6G). Notably, the bio-inspired “pseudo-Neuston” porous network structure shows strong potential for physically capturing microplastics (PS, PET, PMMA) and promoting particle–hotspot contact, thereby enhancing SERS signal acquisition under our laboratory conditions.

To address spectral interference, we proposed a hybrid deep-learning model combining wavelet transform, CNN, and Transformer. This architecture effectively mitigates noise and extracts discriminative features, achieving a classification accuracy of 98.7% under simulated extreme-noise conditions (Gaussian white noise and baseline drift), significantly outperforming traditional machine-learning baselines.

Overall, this study provides a scalable route for fabricating high-performance semiconductor-based SERS substrates and establishes a robust framework for automated spectral analysis. Future work will focus on validating the integrated platform using real environmental water matrices containing naturally occurring interferents.

## Data Availability

The original contributions presented in this study are included in the article/Supplementary Materials. Further inquiries can be directed to the corresponding author.

## Supplementary Materials

The following supporting information can be downloaded at: https://www.mdpi.com/article/10.3390/ma19010090/s1, Figure S1: Raman spectrum of Rhodamine 6G (R6G) on the composite substrate (10^−13^ M); Figure S2: SEM images of AgNPs combined with microplastics; Figure S3. Pore size distribution histogram of the Ta_2_O_5_/AgNPs composite substrate; Figure S4: Random laser scattering intensity of spherical Ta_2_O_5_; Figure S5: Complete training loss, validation loss, and validation accuracy curves; Figure S6: Performance comparison (Accuracy, F1-score) and probability distributions of different models; Figure S7: Confusion matrices of SVM, KNN, Random Forest, and LDA; Figure S8. Simulated electric-field enhancement distribution at 532 nm for Ta_2_O_5_, AgNPs, and the Ta_2_O_5_/AgNPs composite. Table S1: Detailed structure and hyperparameter settings of the model. Table S2. Class-wise performance metrics (Precision and Recall) with standard.
