# Supplementary material for: Synergistically Enhanced Ta2O5/AgNPs SERS Substrate Coupled with Deep Learning for Ultra-Sensitive Microplastic Detection
Source: Materials (Basel). 2025 Dec 25;19(1):90. doi: 10.3390/ma19010090 (PMC12786570; doi:10.3390/ma19010090)
Supplement: Supplementary file 1 [file materials-19-00090-s001.zip › materials-4048144-supplementary.pdf]

Preparation Method:

Silver nanoparticles were synthesized by a chemical method. First, a certain amount of ethylene glycol was added to a 20-milliliter round-bottom flask, which was then placed on a magnetic stirrer and stirred. Subsequently, 0.25 grams of PVP was slowly added and stirred until the solution became clear. In a 5-milliliter test tube, 1 milliliter of deionized water and 0.05 grams of silver nitrate were added and stirred until completely dissolved. The round-bottom flask was placed in an oil bath and rapidly heated. When the temperature reached 120 °C, the prepared silver nitrate solution was quickly added and stirred evenly. The mixture was continuously heated for 40 minutes until the solution turned into a milk tea color. After taking out the solution and cooling it, 40 milliliters of acetone was added for washing, and then centrifugation was carried out. After multiple washing and centrifugation steps, a silver nanoparticle solution was finally obtained.

The Ta_2O_5 nanostructure was prepared by a two-step hydrothermal method. In the first step, 1 gram of tantalum oxide powder was dissolved in 10 milliliters of hydrofluoric acid and placed in a PTFE reaction kettle to react at 110 °C for 12 hours. After cooling, about 20 milliliters of ammonia water was slowly added to adjust the pH to around 9 until a large amount of white precipitate was formed. The precipitate was washed and dried to obtain the Ta_2O_5 precursor. In the second step, under the condition of stirring, the precursor was dissolved in a mixed solution of 30 milliliters of hydrogen peroxide and ammonia water with a volume ratio of 5:1. Then, 0.1 milliliter of hydrofluoric acid was slowly added, and the reaction was carried out at 240 °C for 24 hours. Finally, a white Ta_2O_5 nanoparticle solution was obtained through washing.

Explanation of the Peak Values:

The main characteristic peaks of PS are located at 999 cm^{-1} (symmetrical stretching vibration of the benzene ring) and 1597 cm^{-1} (stretching vibration of the C=C bond in the benzene ring), which reflect its aromatic structure. In addition, the peaks at 624 cm^{-1}, 1030 cm^{-1} and 1205 cm^{-1} correspond to the out-of-plane and in-plane vibrations of the benzene ring, and the peak at 1451 cm^{-1} is related to the bending vibrations of the CH_2 and CH_3 groups. The characteristic peaks of PMMA include 1736 cm^{-1} (stretching vibration of the C=O bond in the ester group) and 1460 cm^{-1} (vibration of the CH_3 group), indicating the presence of ester groups and methyl groups in its molecules. The peaks at 600 cm^{-1} and 969 cm^{-1} correspond to the bending vibration of the C-C-O bond and the stretching vibration of the C-C bond in the main chain, respectively. The main characteristic peaks of PET are 1726 cm^{-1} (stretching vibration of the C=O bond in the ester group) and 1611 cm^{-1} (stretching vibration of the C=C bond in the benzene ring). Other peaks (such as 630 cm^{-1}, 857 cm^{-1} and 1291 cm^{-1}) reflect the presence of aromatic rings and methylene groups.

Table S1. Detailed structure and hyperparameter settings of the model.

Module	Layer/Component	Configuration/Parameters
Preprocessing	Wavelet Transform	Morlet wavelet (Scales: 38–310, Step: 3)
CNN Feature Extractor	Convolution 1	Filters: 384, Kernel: 1×11, Activation: ReLU
	Max Pooling	Kernel: 3×2
	Convolution 2	Filters: 384, Kernel: 1×3, Activation: ReLU
	Convolution 3	Filters: 224 (Embed Size), Kernel: 1×3, Activation: ReLU
Transformer Encoder	Encoder Blocks	Count: 4
	Multi-Head Attention	Heads: 8

	Feed Forward Expansion	Factor: 3
	Dropout	Rate: 0.1
MLP Classifier	Linear Layer 1	Neurons: 4096, Activation: ReLU
	Dropout	Rate: 0.33
	Linear Layer 2	Neurons: 4096, Activation: ReLU
	Output Layer	Neurons: 3 (Classes: PS, PMMA, PET)
Training Settings	Optimizer	Adam
	Loss Function	CrossEntropyLoss
	Learning Rate	0.0003
	Batch Size	64
	Epochs	200

Table S2. Class-wise performance metrics (Precision and Recall) with standard.

	Precision	Precision Err	Recall	Recall Err
PS	0.9870	0.0098	0.9780	0.0085
PMMA	0.9746	0.0110	0.9601	0.0115
PET	0.9708	0.0118	0.9674	0.0112

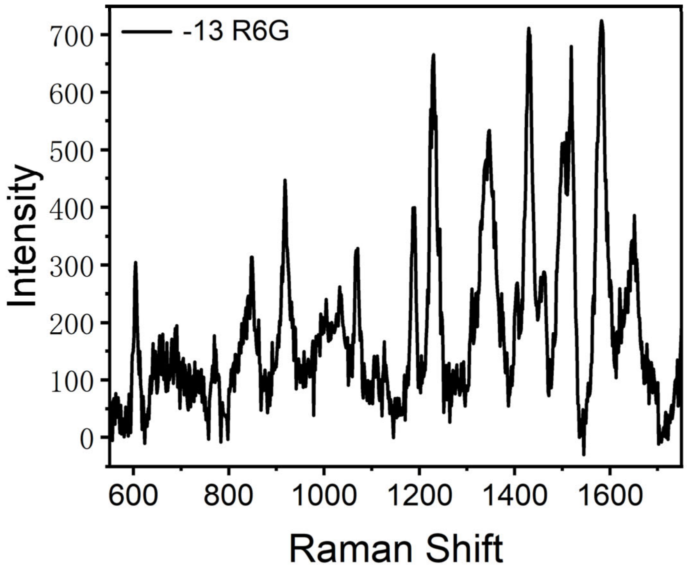

Figure S1. Raman spectrum of R6G on the composite substrate (10^{-13} M) .

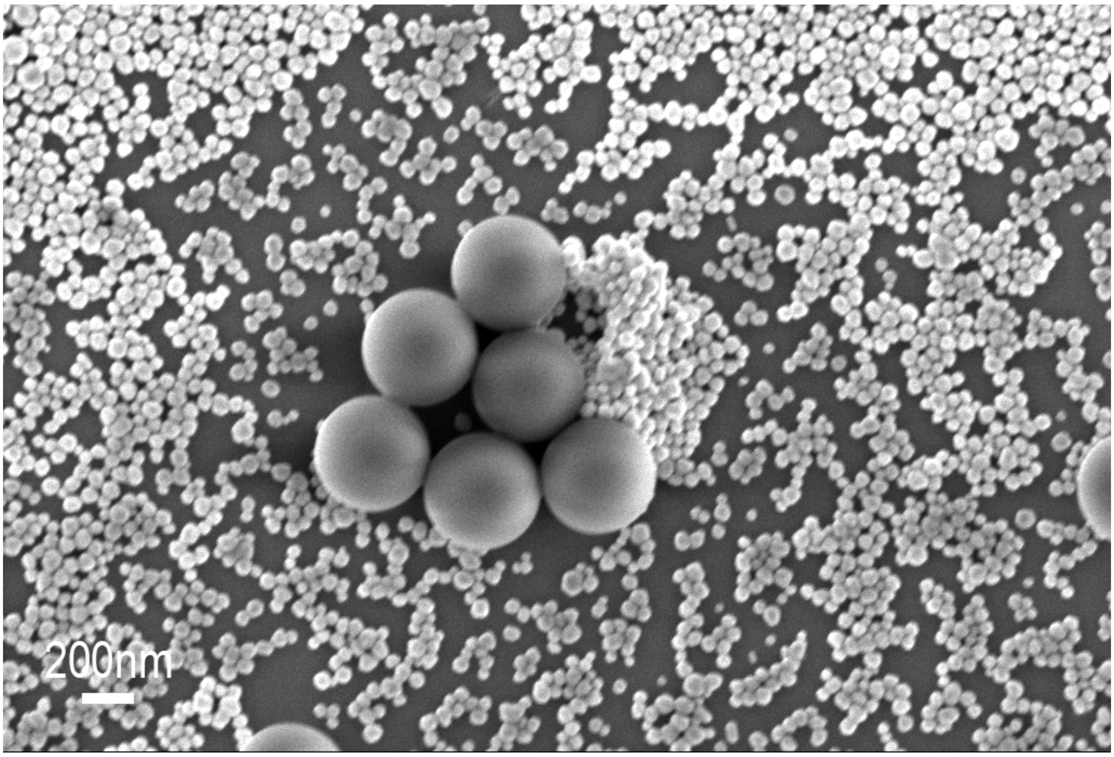

Figure S2. SEM images of AgNPs combined with microplastics.

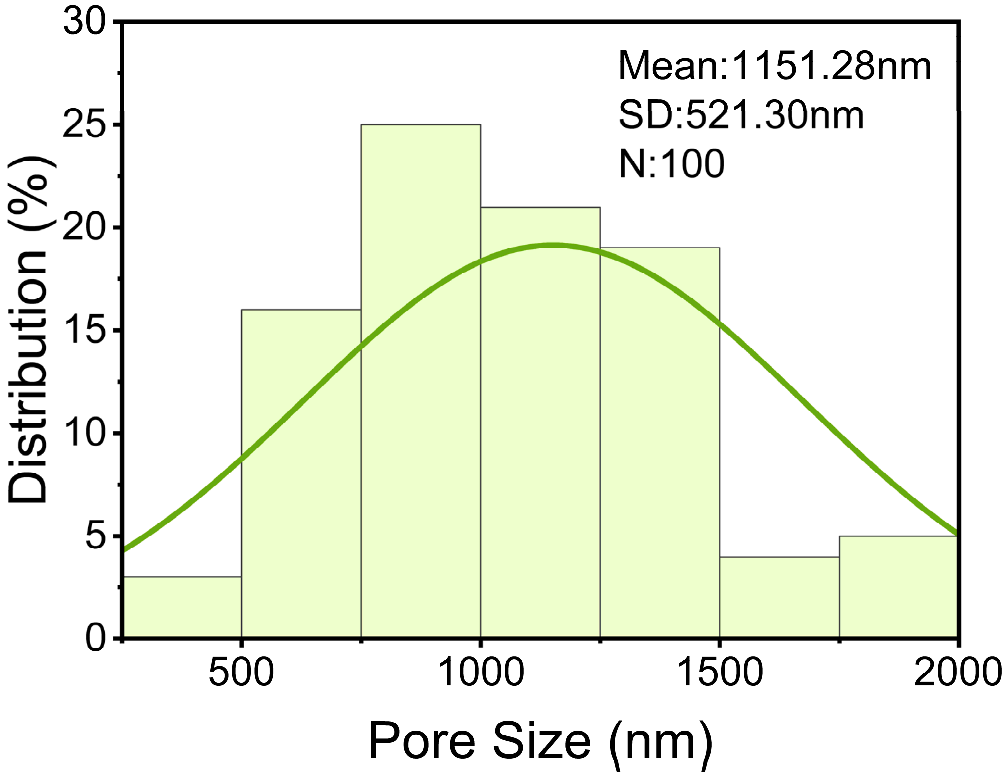

Figure S3. Pore size distribution histogram of the Ta_2O_5/AgNPs composite substrate.

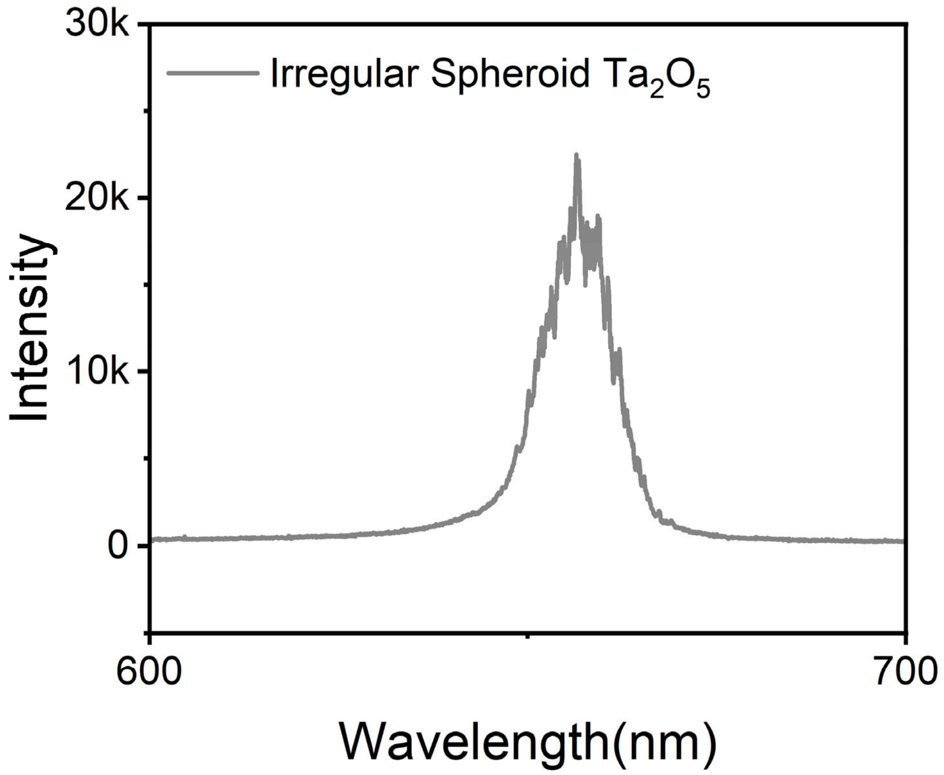

Figure S4. Random laser scattering intensity of spherical Ta_2O_5.

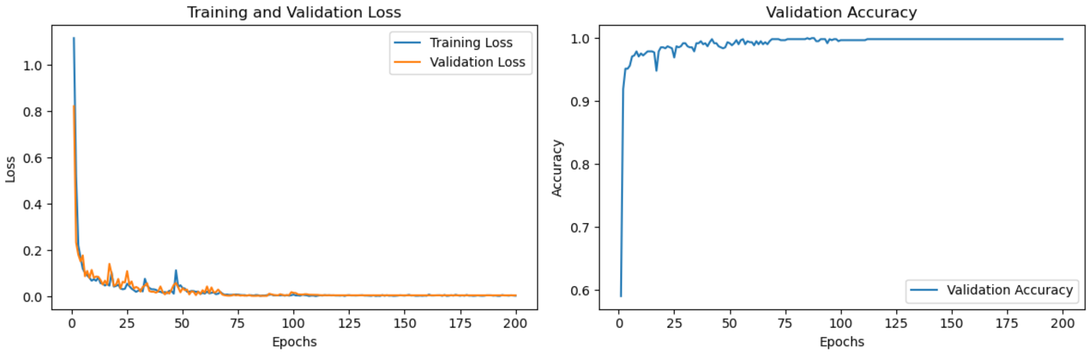

Figure S5. Training loss, validation loss, and validation accuracy curves.

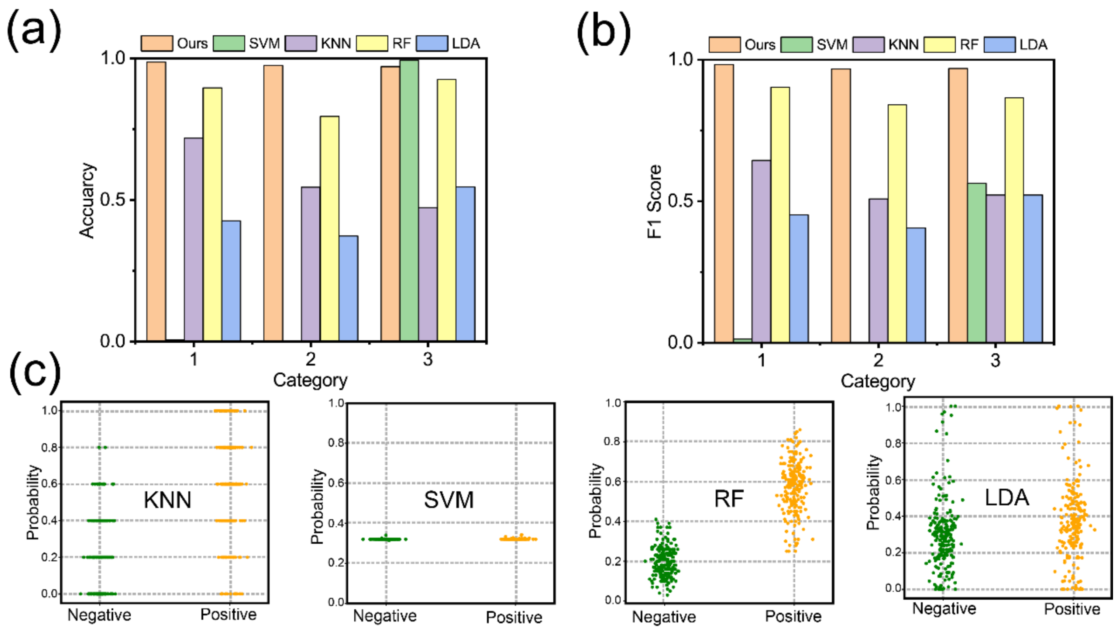

Figure S6. (**a**,**b**) Accuracy and F1-score comparisons among different models; (**c**) Probability distributions from model outputs.

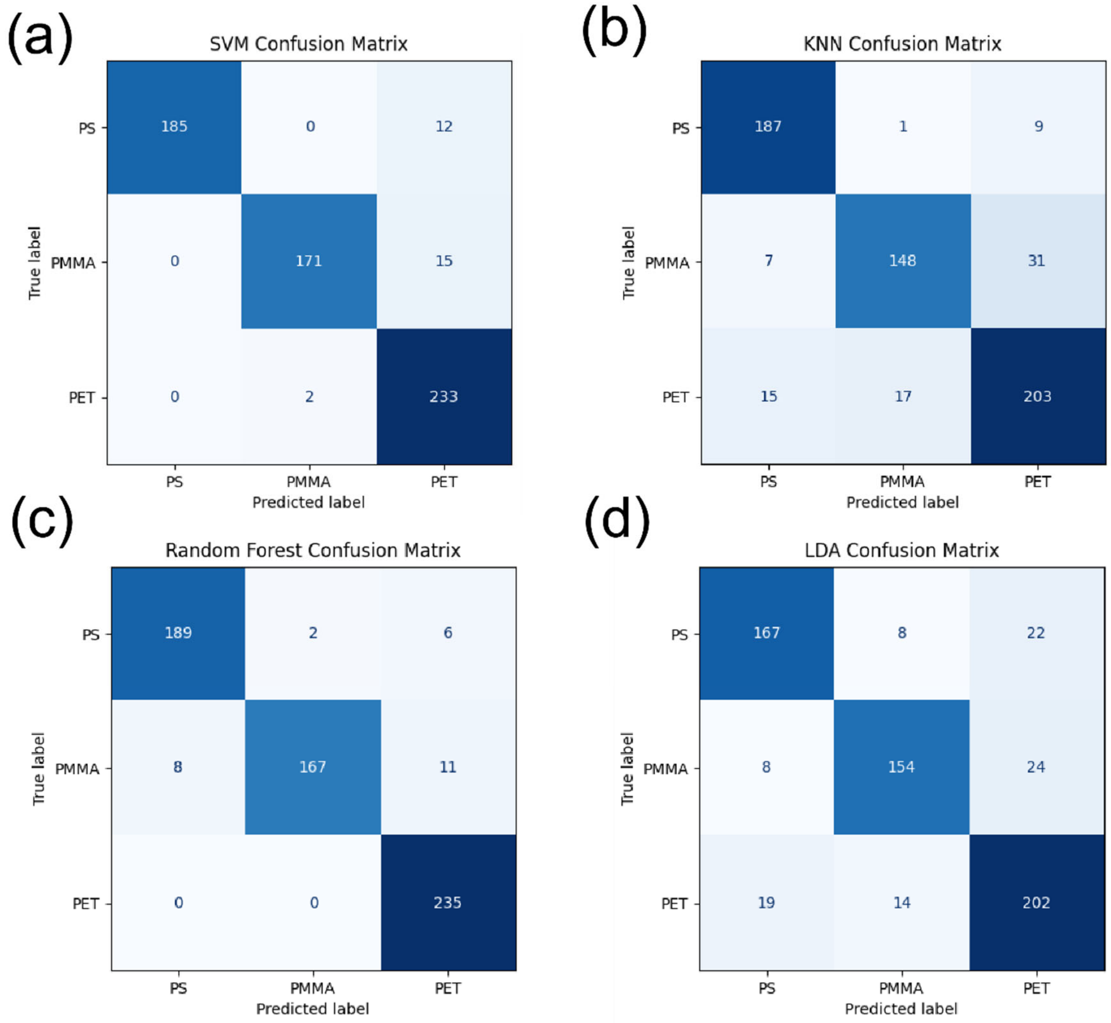

Figure S7. Confusion matrices of (**a**) SVM, (**b**) KNN, (**c**) Random Forest, and (**d**) LDA.

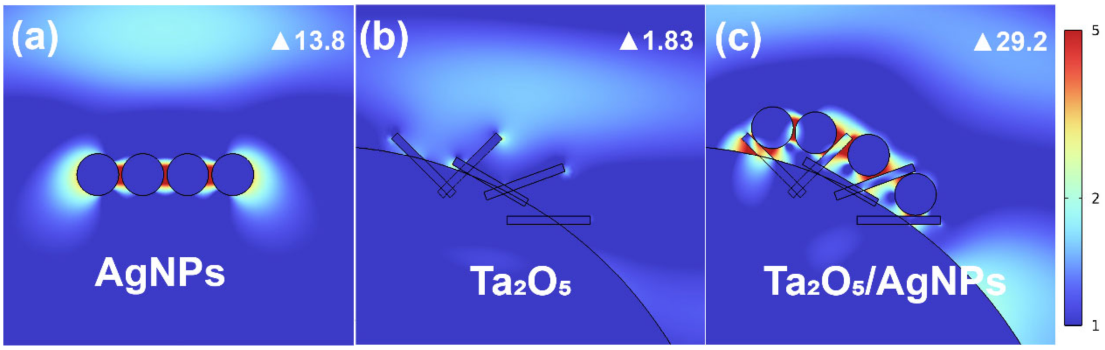

Figure S8. Simulated electric-field enhancement distribution at 532 nm for (**a**) AgNPs, (**b**) Ta_2O_5, and (**c**) the Ta_2O_5/AgNPs composite.
